# Manufacturing Epidemics: The Role of Global Producers in Increased Consumption of Unhealthy Commodities Including Processed Foods, Alcohol, and Tobacco

**DOI:** 10.1371/journal.pmed.1001235

**Published:** 2012-06-26

**Authors:** David Stuckler, Martin McKee, Shah Ebrahim, Sanjay Basu

**Affiliations:** 1 Department of Sociology, University of Cambridge, Cambridge, United Kingdom; 2 Department of Public Health and Policy, London School of Hygiene & Tropical Medicine, London, United Kingdom; 3 South Asian Chronic Disease Network, Public Health Foundation of India, New Delhi, India; 4 Department of Medicine, University of California San Francisco, San Francisco, California, United States of America; 5 Division of General Internal Medicine, San Francisco General Hospital, San Francisco, California, United States of America

## Abstract

In an article that forms part of the *PLoS Medicine* series on Big Food, David Stuckler and colleagues report that unhealthy packaged foods are being consumed rapidly in low- and middle-income countries, consistent with rapid expansion of multinational food companies into emerging markets and fueling obesity and chronic disease epidemics.

**Summary Points**

- The rate of increase in consumption of “unhealthy commodities” (soft drinks and processed foods that are high in salt, fat, and sugar, as well as tobacco and alcohol) is fastest in low- and middle-income countries (LMICs), with little or no further growth expected in high-income countries (HICs).
- The pace at which consumption is rising in LMICs is even faster than has occurred historically in HICs.
- Multinational companies have now achieved a level of penetration of food markets in middle-income countries similar to what they have achieved in HICs.
- Higher intake of unhealthy foods correlates strongly with higher tobacco and alcohol sales, suggesting a set of common tactics by industries producing unhealthy commodities.
- Contrary to findings from studies undertaken several decades ago, urbanisation no longer seems to be a strong risk factor for greater consumption of risky commodities at the population level, with the exception of soft drinks.
- Rising income has been strongly associated with higher consumption of unhealthy commodities within countries and over time, but mainly when there are high foreign direct investment and free-trade agreements. Economic growth does not inevitably lead to higher unhealthy-commodity consumption.

*This article was commissioned for the* PLoS Medicine *series on Big Food that examines the activities and influence of the food and beverage industry in the health arena.*

## Introduction

“Unhealthy commodities”—soft drinks and processed foods that are high in salt, fat, and sugar, as well as tobacco and alcohol—are leading risk factors for chronic noncommunicable diseases (NCDs). Their consumption is thought to be rising rapidly, particularly in LMICs [1]. However, the extent of and reasons for this growth in unhealthy commodity consumption are not well understood.

Many epidemiologists have argued that economic development pushes populations through a “nutrition transition” from undernutrition to overnutrition, shifting food preferences from traditional diets characterised by low salt, saturated fat, and glycaemic indexes to less healthy, complex western diets that lead to obesity and associated NCDs [2]. It has thus been suggested that economic growth and the resulting rising incomes are increasing the risks of unhealthy commodity consumption. Yet studies have also found evidence of the “two faces of malnutrition” [3]: obesity and undernutrition co-occurring in the same households. Poor nutrition among impoverished groups can result in intake of both insufficient nutrition and excess calories (particularly from cheap, non-nutritious foods) [4]. There is also a “social transition” in obesity and consumption of unhealthy foods, as risks initially most prevalent among the wealthiest shift to and become embedded among the lowest-income groups [5],[6],[7]. Paradoxically, these findings indicate that poverty, not higher income, may be a key risk factor for consumption of unhealthy commodities.

To understand why people are choosing to consume unhealthy commodities, it is necessary to study the transformations to economic and social systems that are favouring their increasing availability and affordability. Previous research had focused on the role played by urbanisation in the nutrition transition [8],[9],[10],[11], but with the global rise of transnational food and drink companies there is a clear need to focus on the role of global producers in manufacturing and marketing the commodities implicated in NCD epidemics.

Unhealthy commodities are highly profitable because of their low production cost, long shelf-life, and high retail value. These market characteristics create perverse incentives for industries to market and sell more of these commodities. Coca-Cola's net profit margins, for example, are about one-quarter of the retail price, making soft drink production, alongside tobacco production, among the most profitable industrial activities in the world. Indeed, transnational corporations that manufacture and market unhealthy food and beverage commodities, including Coca-Cola, PepsiCo, and Cadbury Schweppes, are among the leading vectors for the global spread of NCD risks [12],[13],[14]. Increasingly, they target developing countries' markets as a major area for expansion [15],[16],[17].

Neoliberal policies, including the opening of markets to trade and foreign investment, create environments that are conducive to the widespread distribution of unhealthy commodities by multinational firms. A theory of “dietary dependency” [6] proposes that integration into the global economy makes country's food systems come to depend on imports from and investments by large multinational processed food firms. When this happens in LMICs, their populations' consumption choices and habits are increasingly affected by shifts in food type, price, availability, and marketing that favour unhealthy commodities [18]. Reports suggest that when LMIC farmers and food sellers cannot compete with multinational firms, they often collapse or are integrated into processed food production [6],[19].

Although preliminary evidence suggests the linkage of consumption of unhealthy commodities and systems of food trade and market integration, a systematic and global examination of this relationship is needed. In addition, debate has focused largely on HICs, neglecting the pace and scale at which food systems in LMICs are incorporating more unhealthy commodities. While studies have begun to document individual-level risk factors for consumption of such commodities (e.g., socioeconomic status, urban/rural residence, education level) [7], relatively few [20] have assessed the underlying population-wide reasons for the variations across populations in the pace and degree of these dietary transformations among LMICs using quantitative data.

We begin by examining two main questions: (1) Where is the consumption of unhealthy commodities rising most rapidly? and (2) What determines the pace and scale of these increases? For comparison, we analyse data on global trends in tobacco and alcohol commodities. We conclude by identifying policy interventions that could shift dietary patterns in a healthier direction and making recommendations for future research.

## Methods

To describe trends in unhealthy food, beverage, and tobacco commodities, we collected market data on commodity sales from EuroMonitor Passport Global Market Information database 2011 edition, covering up to 80 countries between 1997 and 2010 with forecasts to the year 2016 [21]. Data include both per capita volumes for packaged foods, including snacks, snack bars, ice cream, oils and fats, chilled processed food, dried processed food, canned food, soft drinks, hot drinks, and ready-to-eat meals (a grouping sometimes referred to as ‘ultra-processed’ foods with the exception of oils and fats). Industry data on retail sales of alcohol and tobacco were also obtained from EuroMonitor. To correct for differences in the prices of these products across countries, these data were analysed using fixed exchange rates and constant prices for the year 2011.

These official market data, as reported by governments, have similar limitations to other commonly used macroeconomic data such as gross domestic product (GDP) and trade statistics. Additionally, these data capture only sales volumes, which are an imperfect measure of consumption. In particular, sales data may fail to capture important sources of consumption: food and beverage products may be wasted or produced at home [22], and alcohol and tobacco products may be smuggled. However, sales data have arguably greater validity than alternative survey-based measurements, because they are not subject to recall biases from people understating their levels of consumption (particularly problematic with regard to alcohol and tobacco use). Another advantage is that industry data are much more widely available and consistently reported for tracking across countries and over time than are currently available through survey-based measurements.

## Global Trends in Unhealthy Food, Beverage, and Tobacco Commodities

**Figure 1:**
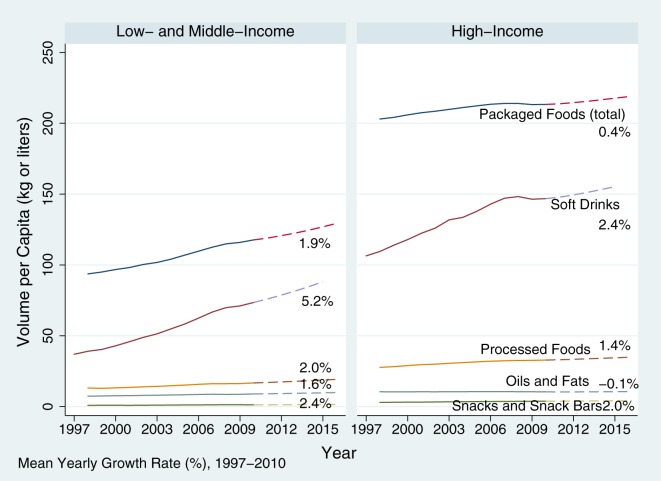
Trends in per capita sales of unhealthy food and beverage commodities, 1997–2010 and projected to 2016. Mean growth rates 1997–2010 are labelled. Data are from the EuroMonitor 2011 dataset. LMICs defined using World Bank criteria as GDP<USD12,500 in the year 2010. Dashed lines are forecast trends between 2011 and 2016.

As a first step, we compared trends in per capita volume of each major food category in LMICs and in HICs, as shown in Figure 1. Based on both average rates of growth between 1997 and 2010 (labelled on the figure) and projected trends from 2011 to 2016 (dashed lines), we can make the following observations (see the figure in Text S1 for disaggregation by geographic region):

***Observation 1*** **. Growth of snacks, soft drinks and processed foods is fastest in LMICs (i.e. GDP≤USD12,500). Little or no growth is expected in HICs in the next 5 years.**

At the current pace of increasing consumption in LMICs and HICs, consumption of unhealthy food commodities will converge with levels currently seen in and projected for HICs within about three decades. Further, as the size of populations in LMICs is more than five times greater than that in HICs, the bulk of unhealthy commodities is already, and will continue to be, consumed in LMIC settings.

The situation with tobacco and alcohol follows a similar pattern, albeit to a less pronounced degree, as shown in Text S2. In HICs, per capita sales of alcohol and tobacco are projected to decline, partly reflecting a short-term dip associated with the economic recessions between 2007 and 2010. In contrast, in LMICs, they are projected to rise by about 20% over the next 5 years and, if current trends are sustained in both groups of countries, would take about four decades to reach the consumption rates of HICs.

Another way to look at the data is to investigate which countries are projected to experience the greatest rises in unhealthy commodities in the next 5 years. The scatterplots in Text S3 disaggregate the regional trends into country-specific patterns for soft drinks and processed foods. As shown in the figure in Text S3, the countries anticipated to have the greatest increases in soft drink consumption per capita include Vietnam and India, where consumption is projected to double, followed by Egypt, China, Tunisia, Cameroon, and Morocco where increases are estimated to be about 50%. These are substantial rises of over 10% per year in population-wide consumption of unhealthy commodities in a very short period of time. To put the speed of these changes in perspective, it is worth noting that consumption of sweeteners and sugary beverages is increasing at a much faster pace than was observed in the United States over the past half century [23],[24].

***Observation 2*** **. The pace of increase in consumption of unhealthy commodities in several LMIC is projected to occur at a faster rate than historically in HICs.**

Which companies are the leading manufacturers and distributors of these commodities? To shed light on this question we investigated the market shares of total packaged foods in Brazil, China, India, Mexico, Russia, and South Africa and, by way of comparison, the United States. As shown in Table 1, in LMICs, multinational corporations have already made a significant entry into food systems. In each country, one of the two market leaders is multinational, with the exception of China. All countries also have Nestle in the top three manufacturers of packaged foods, with the exception of China. Overall, in Brazil Nestle had the highest market share of any multinational company, with 8.4% of the market. In Mexico, PepsiCo and Nestle have market shares of 5.3% and 3.8%, respectively. This level of market concentration is similar to that seen in HICs such as the US, where the leading companies were Kraft (6.8% of market share), PepsiCo (5.2%), and Nestle (4.2%).

**Table 1:** Top 10 manufacturers of packaged foods.

Brazil		China		India		Mexico		Russia		South Africa		USA	
Company	%	Company	%	Company	%	Company	%	Company	%	Company	%	Company	%
Nestlé SA	8.4	China Mengniu Dairy Co Ltd	4.9	Gujarat Co-operative Milk Marketing Federation Ltd	7.9	Grupo Bimbo SAB de CV	9.1	Wimm-Bill-Dann Produkty Pitania OAO	4.7	Tiger Brands Ltd	19.5	Kraft Foods Inc	6.8
Brf Brasil Foods SA	5.0	Inner Mongolia Yili Industrial Group Co Ltd	4.7	Britannia Industries Ltd	5.0	PepsiCo Inc	5.3	Danone, Groupe	4.3	Pioneer Food Group Ltd	6.3	PepsiCo Inc	5.2
Kraft Foods Inc	3.9	Kuok Oils & Grains Pte Ltd (KOG)	3.5	Nestlé SA	4.9	Nestlé SA	3.8	Nestlé SA	2.8	Nestlé SA	4.7	Nestlé SA	4.2
Unilever Group	3.3	Ting Hsin International Group	3.1	National Dairy Development Board	4.8	Industrial Lala SA de CV, Grupo	3.6	Obiedinenye Konditery UK OOO	2.3	Clover Ltd	4.7	Mars Inc	3.2
Groupe Danone	2.8	Shineway Group	2.9	Parle Products Pvt Ltd	4.8	Kraft Foods Inc	2.8	Mars Inc	2.1	Parmalat Group	4.6	Kellogg Co	2.7
PepsiCo Inc	2.5	Hangzhou Wahaha Group	2.2	Kraft Foods Inc	3.1	Ganaderos Productores de Leche Pura SA	2.1	Kraft Foods Inc	1.7	Unilever Group	4.4	General Mills Inc	2.7
Bunge Ltd	2.0	Want Want Group	2.0	Karnataka Cooperative Milk Producers Federation Ltd	2.8	Sigma Alimentos SA de CV	1.8	Unilever Group	1.2	Dairybelle (Pty) Ltd	4.0	Hershey Co, The	2.3
M Dias Branco SA Indústria e Comércio de Alimentos	1.7	Bright Food (Group) Co Ltd	1.6	GlaxoSmithKline Plc	2.7	Kellogg Co	1.7	Valio Oy	1.1	Kraft Foods Inc	3.4	ConAgra Foods Inc	2.1
Private Label	1.6	China National Cereals, Oils & Foodstuffs Imp & Exp Corp (COFCO)	1.4	ITC Group	2.4	Unilever Group	1.7	Cherkizovsky APK	0.9	AVI Ltd	3.3	Unilever Group	2.0
Itambé SA	1.5	Mars Inc	1.3	PepsiCo Inc	2.3	Conservas La Costeña SA	1.1	Yug Rusi APG	0.9	PepsiCo Inc	2.4	Campbell Soup Co	1.6

***Observation 3*** **. Multinational companies have already entered food systems of middle-income countries to a similar degree observed in HICs.**

### Population Determinants of Unhealthy Food, Beverage, and Tobacco Commodities

As Geoffrey Rose famously noted, to understand the reasons for sick populations, one must look not just to individual factors but also societal ones [25]. Why are unhealthy commodities manufactured by both multinational and domestic companies penetrating markets in LMICs? To investigate the population determinants of exposure to unhealthy commodities, we collected data on economic growth, urbanization, and market integration from the World Bank World Development Indicators 2011 edition [26].

**Figure 2:**
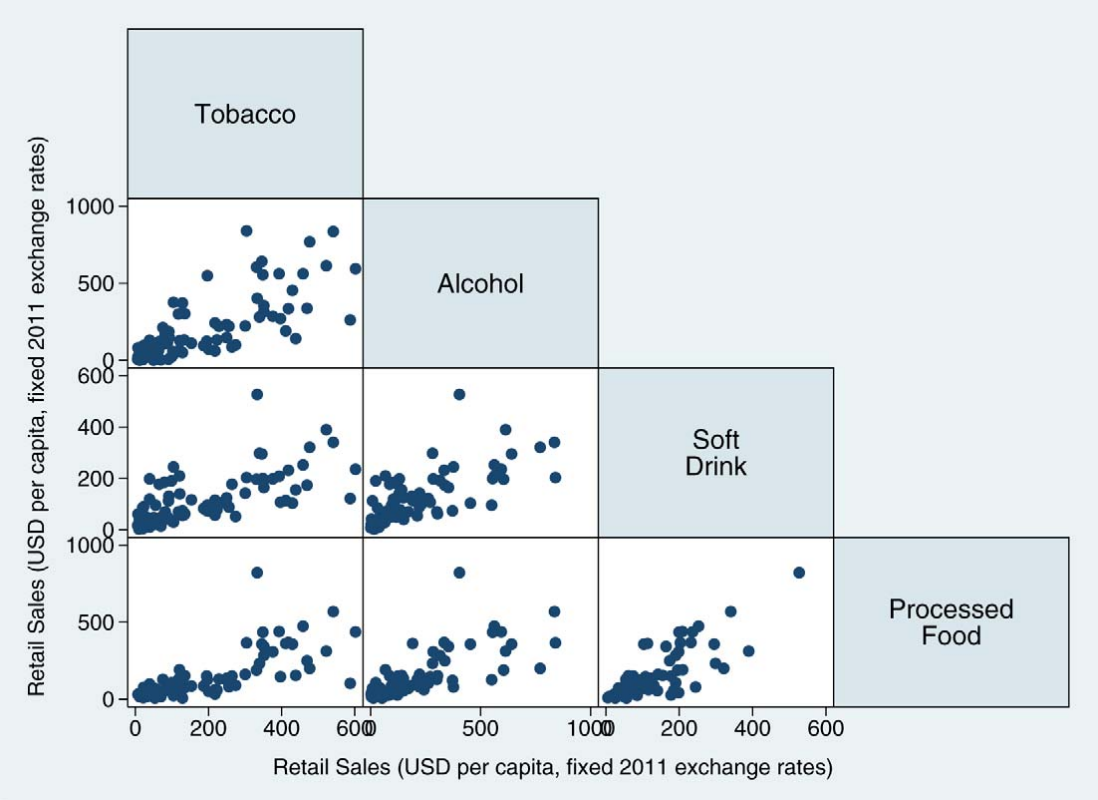
Associations of tobacco, alcohol, soft drink and processed food markets, 80 countries, 2010.

One clue about the underlying causes of this market penetration is the observation that population consumption of unhealthy *non-food* commodities such as tobacco and alcohol are strongly correlated with unhealthy food commodity consumption, as shown in Figure 2. In other words, in countries where there are high rates of tobacco and alcohol consumption, there is also a high intake of snacks, soft drinks, processed foods, and other unhealthy food commodities. The correlations of these products with unhealthy foods suggest they share underlying risks associated with the market and regulatory environment.

***Observation 4*** **. Tobacco and alcohol are joint risks with unhealthy food commodities.**

Economic development is often argued to be the main factor explaining the rising intake of unhealthy commodities in LMICs. This view garners support from the food commodity data. There was a moderately strong association of greater GDP per capita with consumption rates of soft drinks (*r* = 0.59, *p*<0.0001), snacks (*r* = 0.71, *p*<0.0001), processed foods (*r* = 0.66, *p*<0.0001), alcohol volume (*r* = 0.48, *p*<0.0001), and tobacco sales (*r* = 0.79, *p*<0.0001), but not oils and fats (*r* = 0.05, *p* = 0.65) in the year 2010.

***Observation 5*** **. Substantial increases in consumption of unhealthy commodities are not an inevitable consequence of economic growth.**

Several countries do not follow the general correlation between per capita GDP and consumption in each food category, indicating that increased consumption of unhealthy commodities is not an inevitable result of economic development. The figure in Text S4 depicts the correlation between per capita soft drink consumption and GDP across 76 countries for the year 2010. South Korea, Finland, and Sweden have relatively low consumption of soft drinks per capita for their level of GDP, roughly equivalent to countries with about one-third the size of their economies such as Brazil. In contrast, Mexico is a clear outlier (followed by Argentina); its soft drink consumption exceeds that of any other country in the dataset, with the average person consuming more than 300 litres of soft drinks per year. Mexico also has the highest rate of child obesity in developing countries (>30% prevalence), second only to the US. Similar variations related to social and economic policies can be seen for trends in tobacco consumption, as described in greater detail in Text S5.

To analyse further the relationships among per capita income, market integration, and consumption of unhealthy commodities, we used statistical models of populations' consumption over time in 50 LMICs (GDP≤USD12,500 USD). Table 2 shows the results of ten models of the determinants of unhealthy food commodities, illustrating the following main points:

**Table 2:** Determinants of the level of population exposure to unhealthy foods, beverages, and tobacco, 44 LMICs (GDP<USD12,500 per capita), 1997–2010.

Covariate	Snacks (kg per capita)	Confections (kg per capita)	Soft drinks (litre per capita)	Ice cream (kg per capita)	Oils and fats (kg per capita)	Ready meals (kg per capita)	Processed foods (kg per capita)	Packaged foods (kg per capita)	Tobacco (USD sales per capita)	Alcohol (USD sales per capita)
Log GDP per capita (constant USD, purchasing power parity)	0.81* (0.31)	1.60*** (0.28)	42.8*** (11.4)	0.82** (0.26)	2.89*** (0.78)	0.76** (0.22)	6.94*** (1.34)	26.9** (8.61)	63.8*** (13.5)	23.0** (6.73)
Foreign Direct Investment as a % of GDP	0.0085* (0.0036)	0.0085 (0.0046)	0.71** (0.21)	0.0011 (0.0034)	−0.013 (0.019)	0.0017 (0.0039)	0.061** (0.019)	0.27 (0.15)	1.34 (0.68)	0.24* (0.10)
Percentage of Population Living in Urban Settings	0.024 (0.021)	−0.025 (0.019)	2.20* (0.84)	−0.0087 (0.014)	0.043 (0.077)	−0.027* (0.012)	0.16 (0.21)	1.78 (1.17)	−2.21 (1.22)	−0.41 (0.34)
Number of Country-Years	341	560	609	560	560	489	472	560	609	609
Number of Countries	40	49	50	49	49	45	43	49	50	50
*R* ^2^	0.369	0.495	0.483	0.235	0.278	0.345	0.509	0.311	0.216	0.411

*Notes:* Robust-clustered errors in parentheses to reflect non-independence of country sampling.

*: *p*<0.05,

**: *p*<0.01,

***: *p*<0.001.

1. Rising income levels is a significant correlate of increasing exposure to unhealthy foods among low- and lower–middle-income countries
2. Contrary to the findings from research conducted in the past, urbanization is no longer a significant correlate of exposure to unhealthy foods (with the exception of soft drinks)
3. Alternatively, greater market integration, as indicated by higher levels of foreign direct investment as a fraction of GDP, is a strong correlate of greater exposure to unhealthy food commodities, especially for soft drink, processed foods, and alcohol.

The discrepancy with earlier research on urbanization is not surprising, given the strenuous efforts undertaken over recent decades by transnational food and drink corporations to ensure penetration of their products into rural areas, a development now being taken advantage of in a range of partnerships to distribute antiretrovirals and condoms (Table 2) [27].

***Observation 6.*** **Foreign direct investment increases risks of rising unhealthy commodities among LMICs.**

Additionally, we investigated whether countries with greater levels of foreign direct investment as a fraction of GDP, reflecting greater foreign corporate entrance into countries' domestic economic system, modified the effects of the association of rising GDP with population-wide consumption of unhealthy food commodities. As shown in the tables of Text S6, in periods when foreign direct investment was relatively low (<2% of GDP), there was no significant association between GDP and confectionery, ice cream, processed foods, packaged foods, and tobacco; and, in other cases, the effect size tended to diminish, particularly for soft drinks. This suggests that rising incomes with limited penetration by multinational corporations into the domestic economy do not necessarily give rise to higher intake of unhealthy commodities.

As one further test of the dietary dependency hypothesis, we investigated whether LMICs that entered into free-trade agreements with the United States had higher levels of consumption of soft drinks than those that did not, after correcting for the country's level of GDP per capita and urbanization. The table in Text S7 shows that such free-trade agreement is associated with about a 63.4% higher level of soft drink consumption per capita (95% CI: 24.0% to 103.3%).

There is extensive research linking rates of unhealthy commodity consumption with obesity, diabetes, and chronic disease outcomes [28],[29],[30]. We similarly note that there is a strong statistical relationship between consumption of these unhealthy commodities worldwide and population levels of obesity, as shown in the packaged food versus obesity figure in Text S8. As in many cases, such industry data are widely available, they may act as early indicators, so-called ‘leading indicators’, for NCD epidemics.

## Towards a Corporatology of Food, Beverage, and Other Unhealthy Commodities

Taken together, these data show that there is significant penetration by multinational processed food manufacturers such as Nestle, Kraft, PepsiCo, and Danone into food environments in LMICs, where consumption of unhealthy commodities is reaching—and in some cases exceeding—a level presently observed in HICs. Greater population consumption of unhealthy food commodities tends to occur in countries with high tobacco and alcohol consumption, suggesting a set of common tactics by industries producing all unhealthy commodities. Overall, the LMICs experiencing the highest exposure to unhealthy commodities are not just those in which growth is occurring most rapidly, but those in which such development is occurring in the context of food systems that are highly penetrated by foreign multinationals. Several middle- and high-income countries have preserved economic growth without consuming high volumes of unhealthy commodities, suggesting that domestic policy choices may be critical to mitigating future NCD risk. Previous alternative explanations for rising unhealthy commodity consumption implicating demographic changes, such as urbanization, no longer find strong support in the population-level data, apart from a significant association with increasing exposure to soft drinks, plausibly because these products are easier to obtain at low cost in dense, urban settings. However, even this may change in the near future.

There is an increasing understanding of how unhealthy commodities might be regulated. At least three population-wide cases illustrate the potential for countries to improve regulation of food, beverage, and tobacco commodity systems, as shown in the figure in Text S9. First, Mexico, under pressure from the international financial community following its “Tequila crisis in 1994,” rapidly opened its markets to trade and foreign investment by entering a trade agreement with the US. It experienced a rapid rise in soft drink consumption throughout the decade with entry of multinational producers into the market, consistent with the findings reported in Text S7. In contrast, Venezuela, which does not have such an agreement with the US, has maintained steady consumption rates during the 1990s and 2000s, despite experiencing high levels of economic growth (largely from oil). Second, Brazil, despite being the second largest producer of tobacco worldwide, made tobacco control a high priority in the late 1990s [31], including adopting measures to increase the price of cigarettes and ban smoking in public places (prior to the ratification of the Framework Convention on Tobacco Control in 2003) [32]. As shown in the figure in Text S9, Brazil's rate of tobacco consumption fell by 75% between 1998 and 2003 and has since remained at low levels. Meanwhile, Chile, which delayed ratification of the Framework Convention and its implementation, initially had lower levels of tobacco consumption, but steadily experienced increases so that its level is now more than twice as high as Brazil. Third, turning to data from HICs, the United Kingdom, often characterized as a having an excessive “drinking culture,” actually did not have high levels of alcohol consumption per capita in the late 1990s (about 30% lower than in France). However, steady increases throughout the past decade under the Labour government, which introduced a range of deregulatory policies associated with easier access and cheap sales by large supermarkets, have reversed the situation so that now consumption is about 30% higher than in France.

Further research is needed in several areas to address the rise in consumption of unhealthy commodities and its associated health consequences. There is a clear need for better data to enable investigation of underlying drivers of consumption of unhealthy food and beverages, and of tobacco commodities—including retail food sector-specific foreign direct investment; accession to free-trade agreements; tariff and import duties; market concentration of transnational corporations; the percentage of retail space owned by a limited number of firms; market regulations and protections; and the capacity to produce and distribute low-cost healthy alternatives domestically. Our analysis found some evidence that free-trade agreements with the US are linked to greater consumption of soft drinks, even after correcting for the trading partner's level of income per capita. Free-trade agreements typically limit trade and market restrictions on imports of unhealthy commodities and such non-tariff measures as licensing, quotas, prohibitions, bans, and other restrictions having equivalent effect [33],[34]; however, these fiscal policies that increase prices and limit the availability of unhealthy products are among the most effective and low-cost strategies for preventing their consumption. Further studies are needed to test current and prior population-level experiments of trade and capital market integration, the spread of unhealthy commodities, and their links to adverse NCD outcomes.

Research is also needed to find ways to identify more easily conflicts of interest involving companies that make, market, and distribute unhealthy commodities, and that can understand which models of interaction with these companies could orient food systems towards promoting improved nutritional quality and reduced risks of NCDs [35]. This work could link to the development of the Conflicts of Interest Coalition [36] that emerged from the recent advocacy of the NCD Alliance during preparation of the UN High-Level meeting on prevention and control of NCDs. Lastly, most work seeking to mitigate the rise of NCDs focuses on individual behavioural change, including “lifestyle modification” and, in some cases, through medical interventions such as nicotine substitutes or bariatric surgery [6]. While these interventions are highly profitable to pharmaceutical companies and some health professionals, they do little to address the conditions giving rise to consumption of unhealthy commodities and associated NCDs. There is a need to identify population-level social, economic, and political interventions that could stem the rise of unhealthy commodity consumption, and overcome the political barriers to their implementation, as has been done for tobacco control but in which progress remains slow and inadequate in most LMICs.

While it is important to maintain a focus on LMICs, there is also potential for progress by addressing the current and high levels of consumption of unhealthy commodities in HICs. Many transnational companies have made commitments to remove trans fats and reduce levels of salt, sugar, and fat content in foods in wealthy countries. However, in most cases, these nutritional improvements are not being applied in low- and middle-income markets.

NCDs are the current and future leading causes of global ill health; unhealthy commodities, their producers, and the markets that power them, are their leading risk factors. Until health practitioners, researchers, and politicians are able to understand and identify feasible ways to address the social, economic, and political conditions that lead to the spread of unhealthy food, beverage, and tobacco commodities, progress in areas of prevention and control of NCDs will remain elusive.

## Supporting Information

Text S1 Unweighted trends in unhealthy commodities, by geographic region, 2000–2010 and 2010–2015.
(DOC)

Text S2 Trends in tobacco and alcohol commodities, 1997–2010 and projected to 2016.
(DOC)

Text S3 Relationship between projected percentage increase in soft drink consumption and GDP, year 2010–2015, 76 countries.
(DOC)

Text S4 Relationship between per capita consumption of soft drinks and GDP, year 2010, 74 countries.
(DOC)

Text S5 A note on economic growth and tobacco consumption.
(DOC)

Text S6 Replication of Table 2, high- and low-foreign direct investment/GDP.
(DOC)

Text S7 Free-trade agreements and soft drink consumption, 35 LMICs, year 2010.
(DOC)

Text S8 Association of packaged food volume (per capita) with sugar, fat, and salt consumption per capita and obesity and diabetes prevalence, 2005.
(DOC)

Text S9 Three population-level quasi-natural experiments of soft drinks, tobacco, and alcohol consumption.
(DOC)

## Abbreviations

GDP: gross domestic product
HICs: high-income countries
LMICs: low- and middle-income countries
NCD: noncommunicable disease
