# Supplementary material for: Manufacturing Epidemics: The Role of Global Producers in Increased Consumption of Unhealthy Commodities Including Processed Foods, Alcohol, and Tobacco
Source: PLoS Med. 2012 Jun 26;9(6):e1001235. doi: 10.1371/journal.pmed.1001235 (PMC3383750; doi:10.1371/journal.pmed.1001235)

**Supporting Information Text S1. Unweighted Trends in Unhealthy Commodities, by Geographic Region, 2000-2010 and 2010-2015**


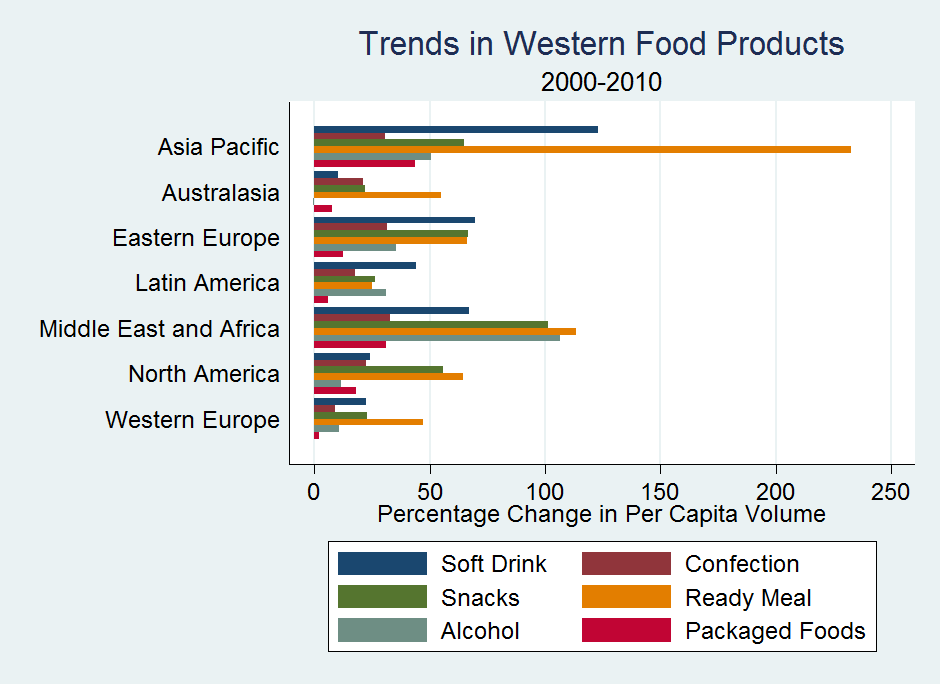

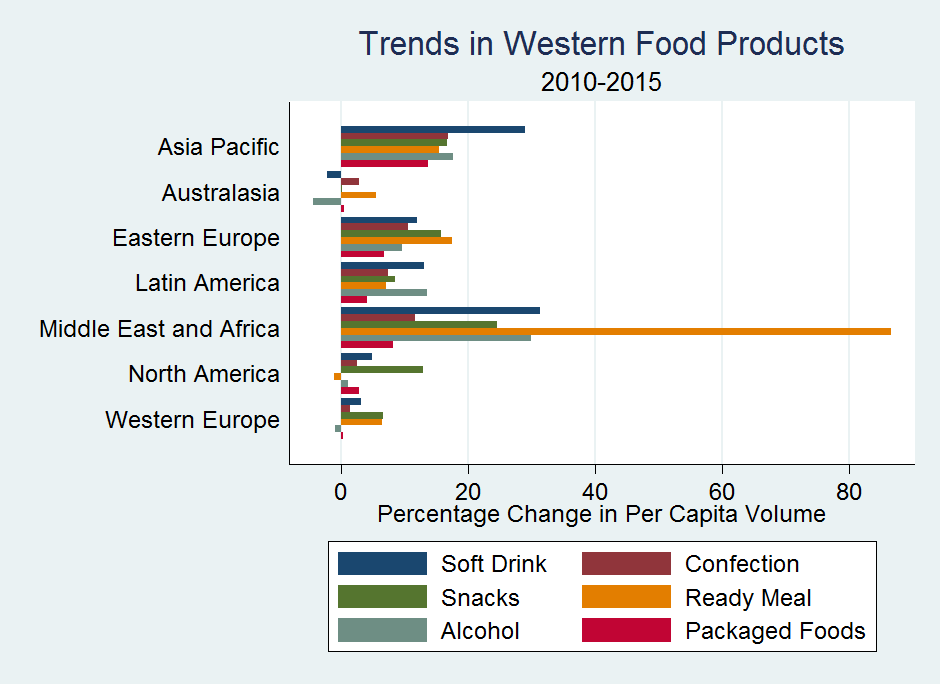

Supplement: Text S1 — Unweighted trends in unhealthy commodities, by geographic region, 2000–2010 and 2010–2015. (DOC) [file pmed.1001235.s001.doc]
