# Supplementary material for: Manufacturing Epidemics: The Role of Global Producers in Increased Consumption of Unhealthy Commodities Including Processed Foods, Alcohol, and Tobacco
Source: PLoS Med. 2012 Jun 26;9(6):e1001235. doi: 10.1371/journal.pmed.1001235 (PMC3383750; doi:10.1371/journal.pmed.1001235)
Supplement: Text S2 — Trends in tobacco and alcohol commodities, 1997–2010 and projected to 2016. (DOC) [file pmed.1001235.s002.doc]

**Supporting Information Text S2**

Figure: Trends in Tobacco and Alcohol Commodities, 1997-2010 and projected to 2016


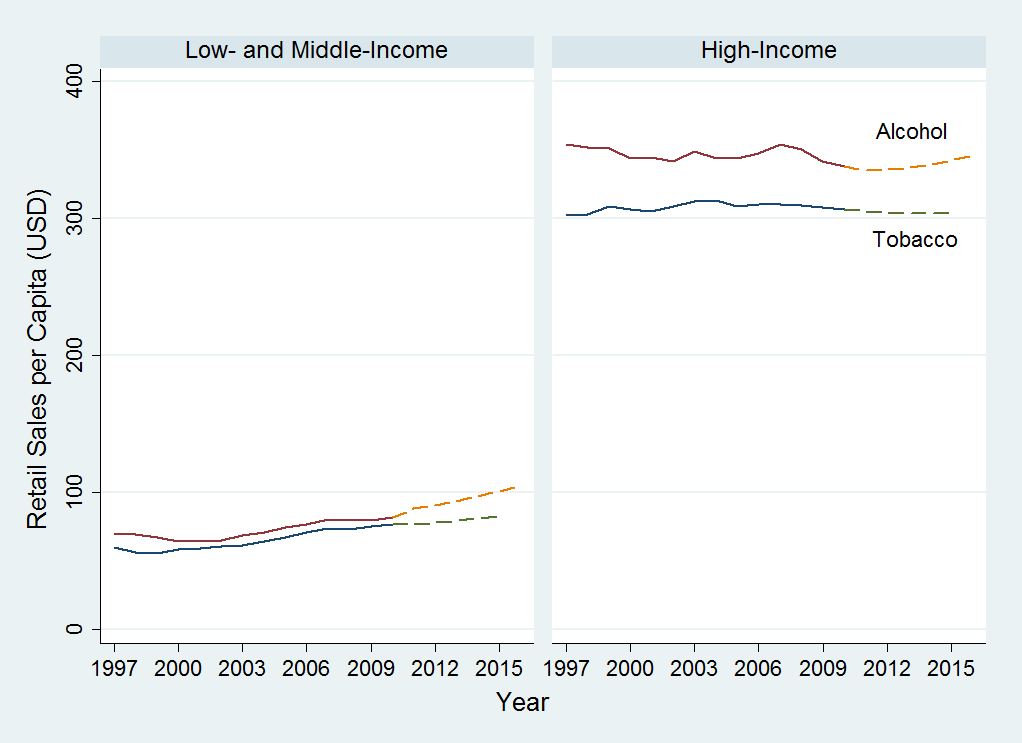


*Notes:* Data are from the EuroMonitor 2011 dataset. Low- and middle-income countries defined using World Bank criteria as GDP <$12,500 in the year 2010. Dashed lines are forecast trends between 2011 and 2016 based on EuroMonitor reports. Data are in constant USD in fixed 2011 exchange rates.
