# Supplementary material for: Manufacturing Epidemics: The Role of Global Producers in Increased Consumption of Unhealthy Commodities Including Processed Foods, Alcohol, and Tobacco
Source: PLoS Med. 2012 Jun 26;9(6):e1001235. doi: 10.1371/journal.pmed.1001235 (PMC3383750; doi:10.1371/journal.pmed.1001235)

**Supporting Information Text S3**

Figure. Relationship between Projected percentage increase in Soft Drink consumption and GDP, year 2010-2015, 76 countries


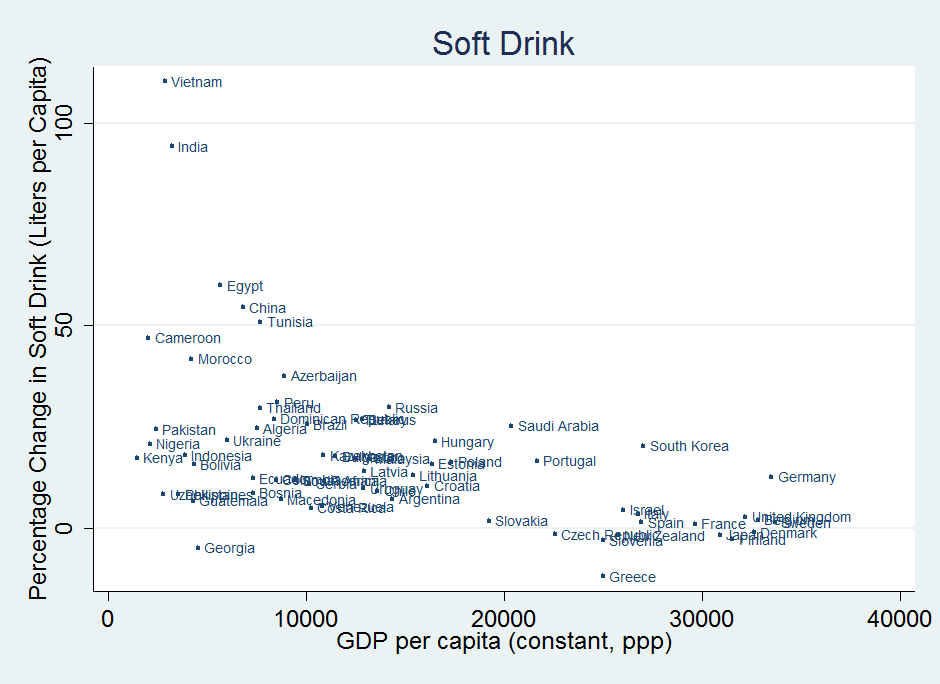

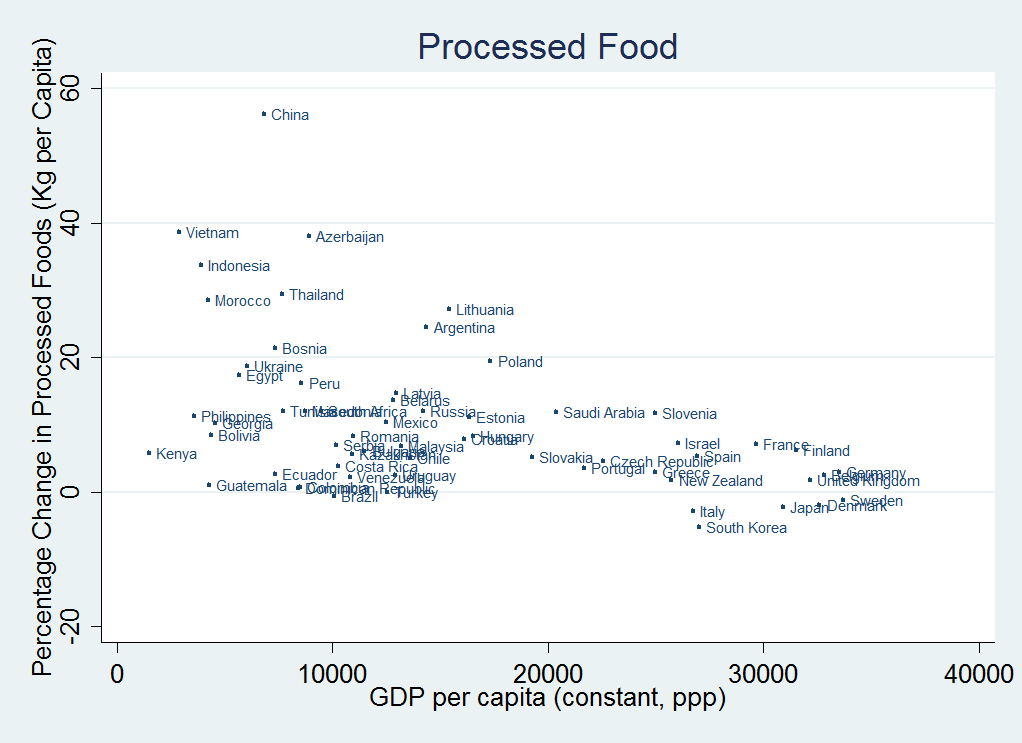

Supplement: Text S3 — Relationship between projected percentage increase in soft drink consumption and GDP, year 2010–2015, 76 countries. (DOC) [file pmed.1001235.s003.doc]
