# Supplementary material for: Manufacturing Epidemics: The Role of Global Producers in Increased Consumption of Unhealthy Commodities Including Processed Foods, Alcohol, and Tobacco
Source: PLoS Med. 2012 Jun 26;9(6):e1001235. doi: 10.1371/journal.pmed.1001235 (PMC3383750; doi:10.1371/journal.pmed.1001235)

**Supporting Information Text S4**

Figure: Relationship between per capita consumption of soft drinks and GDP, year 2010, 74 countries


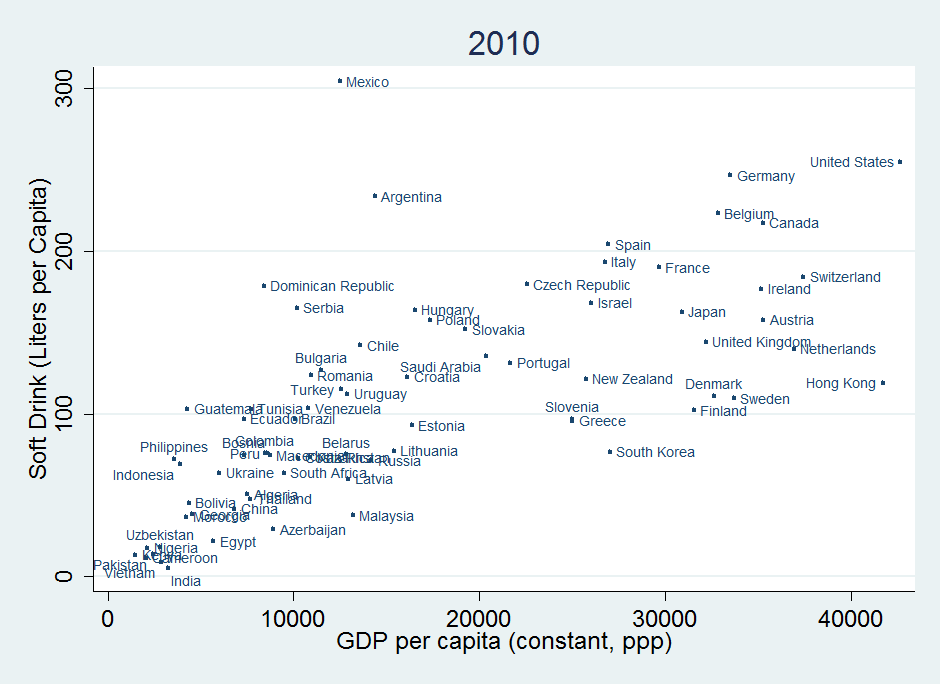

Supplement: Text S4 — Relationship between per capita consumption of soft drinks and GDP, year 2010, 74 countries. (DOC) [file pmed.1001235.s004.doc]
