# Supplementary material for: Manufacturing Epidemics: The Role of Global Producers in Increased Consumption of Unhealthy Commodities Including Processed Foods, Alcohol, and Tobacco
Source: PLoS Med. 2012 Jun 26;9(6):e1001235. doi: 10.1371/journal.pmed.1001235 (PMC3383750; doi:10.1371/journal.pmed.1001235)
Supplement: Text S5 — A note on economic growth and tobacco consumption. (DOC) [file pmed.1001235.s005.doc]

**Supporting Information Text S5. A note on economic growth and tobacco consumption**

The change in tobacco consumption provides further evidence that increases in intake of unhealthy commodities are not the inevitable result of rising incomes, but is conditional upon social and economic policies or other factors. The Framework Convention on Tobacco Control, an international tobacco control treaty, includes a range of policies including tobacco taxes, bans in public spaces, and labeling of tobacco products. The figure depicts the correlation of the average rate of economic growth from 2000 to 2007 with the average growth in tobacco consumption. Brazil and Ecuador, among the early signatory countries to the Convention, stand out for having achieved high rates of real economic growth, averaging about 3% over the past decade, but have actually reduced tobacco consumption by more than 10% per year. In Brazil, tobacco sales per capita have fallen from $185.6 USD in 1997 to $55.2 USD in 2010. This situation contrasts sharply with eastern European countries, particularly Bosnia, Serbia, Uzbekistan, Bulgaria, and Ukraine, where tobacco consumption has grown by over 10% per year. (However in view of concerns about cigarette smuggling in this region these sales rates may be over-estimated because they neither capture exported tobacco, but nor do they captured inward smuggling). Russia, in contrast, has maintained similarly high rate rates but experienced zero growth in tobacco per capita; China is also an intermediate case, with the highest rates of growth in the dataset but tobacco growth of about 5% per year.

Figure: Average Growth in Economic Output and Tobacco Consumption (% per year), 2000-2007, r= 0.51

**
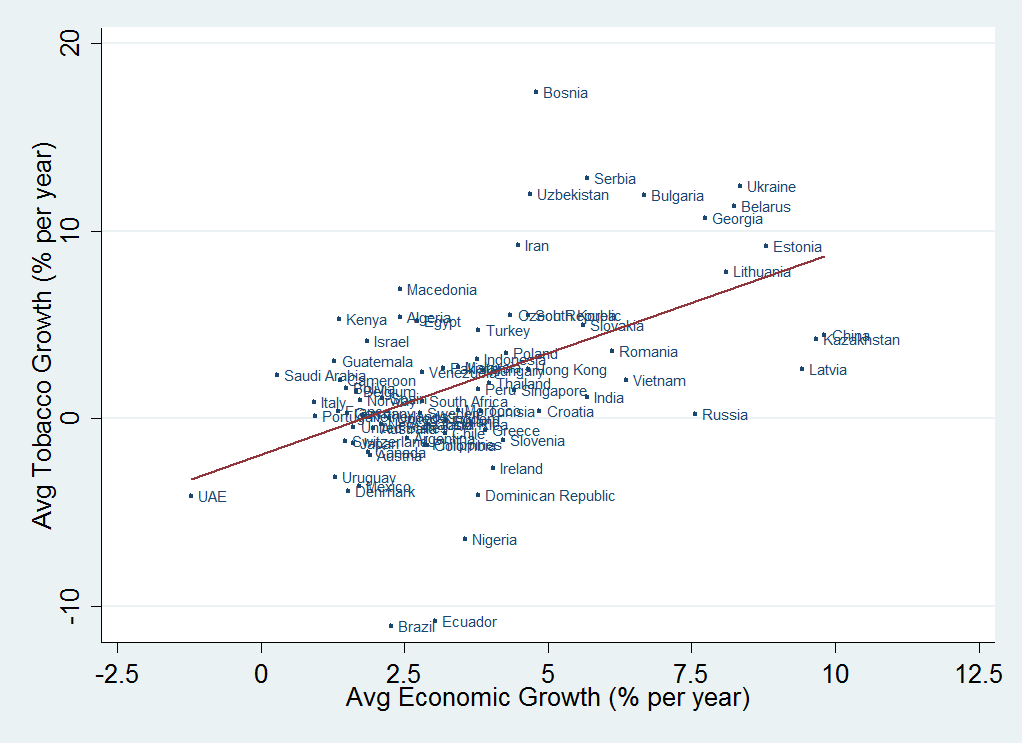
**
