# Supplementary material for: Manufacturing Epidemics: The Role of Global Producers in Increased Consumption of Unhealthy Commodities Including Processed Foods, Alcohol, and Tobacco
Source: PLoS Med. 2012 Jun 26;9(6):e1001235. doi: 10.1371/journal.pmed.1001235 (PMC3383750; doi:10.1371/journal.pmed.1001235)
Supplement: Text S6 — Replication of Table 2, high- and low-foreign direct investment/GDP. (DOC) [file pmed.1001235.s006.doc]

**Supporting Information Text S6: Replication of Table 2, High- and Low-Foreign Direct Investment/GDP**

**Table 1:High Foreign Direct Investment (≥2% of GDP)**

| Covariate | Snacks | Confection | Soft Drink | Ice Cream | Oils and Fats | Ready Meals | Processed Foods | Packaged Foods | Tobacco | Alcohol |
| --- | --- | --- | --- | --- | --- | --- | --- | --- | --- | --- |
|  | (kg per capita) | (kg per capita) | (liter per capita) | (kg per capita) | (kg per capita) | (kg per capita) | (kg per capita) | (kg per capita) | (USD sales per capita) | (USD sales per capita) |
| GDP per capita (constant USD, purchasing power parity) | 1.35 (0.75) | 1.24^*^ (0.58) | 30.3^*^ (13.0) | 0.33 (0.49) | 1.91 (1.44) | 1.18 (0.60) | 6.29 (3.23) | 37.1 (26.2) | 40.0 (25.1) | 27.1^*^ (13.0) |
| Foreign Direct Investment as a % of GDP | -0.0064 (0.0062) | 0.0017 (0.0064) | 0.11 (0.38) | 0.0072 (0.010) | 0.0055 (0.038) | 0.0031 (0.0044) | 0.15 (0.14) | -0.039 (0.58) | -1.85^*^ (0.88) | -0.22^*^ (0.087) |
| Percentage of Population Living in Urban Settings | -0.012 (0.040) | 0.0050 (0.030) | 2.33^**^ (0.78) | 0.016 (0.023) | 0.16 (0.14) | -0.044 (0.027) | 0.50 (0.31) | 3.20 (2.38) | 0.60 (0.89) | -0.63 (0.50) |
| Number of Country-Years | 107 | 205 | 223 | 205 | 205 | 171 | 151 | 205 | 223 | 223 |
| Number of Countries | 28 | 35 | 36 | 35 | 35 | 31 | 30 | 35 | 36 | 36 |
| *R*^2^ | 0.45 | 0.37 | 0.66 | 0.086 | 0.29 | 0.34 | 0.63 | 0.33 | 0.14 | 0.33 |

*Notes:* Standard errors in parentheses

^*^ *p* < 0.05, ^**^ *p* < 0.01, ^***^ *p* < 0.001

**Table 2. Low Foreign Direct Investment (<2% of GDP)**

| Covariate | Snacks | Confection | Soft Drink | Ice Cream | Oils and Fats | Ready Meals | Processed Foods | Packaged Foods | Tobacco | Alcohol |
| --- | --- | --- | --- | --- | --- | --- | --- | --- | --- | --- |
|  | (kg per capita) | (kg per capita) | (liter per capita) | (kg per capita) | (kg per capita) | (kg per capita) | (kg per capita) | (kg per capita) | (USD sales per capita) | (USD sales per capita) |
| GDP per capita (constant USD, purchasing power parity) | 1.07^**^ (0.30) | 1.76^***^ (0.25) | 58.1^***^ (14.1) | 0.74^*^ (0.34) | 3.12^**^ (1.09) | 0.83^**^ (0.24) | 9.05^***^ (1.25) | 29.5^**^ (8.47) | 80.2^***^ (17.1) | 29.9^***^ (5.58) |
| Foreign Direct Investment as a % of GDP | -0.0025 (0.0021) | -0.0014 (0.0038) | 0.24 (0.24) | -0.00068 (0.0034) | 0.0020 (0.018) | -0.0045 (0.0028) | -0.030 (0.015) | -0.084 (0.12) | 1.20 (0.94) | -0.035 (0.069) |
| Percentage of Population Living in Urban Settings | 0.0019 (0.017) | -0.052^*^ (0.022) | 1.12 (1.32) | -0.011 (0.020) | -0.048 (0.092) | -0.032 (0.016) | -0.19 (0.15) | 0.13 (0.71) | -3.99^*^ (1.80) | -0.86^*^ (0.34) |
| Number of Country-Years | 234 | 355 | 386 | 355 | 355 | 318 | 321 | 355 | 386 | 386 |
| Number of Countries | 37 | 49 | 49 | 49 | 49 | 45 | 43 | 49 | 49 | 49 |
| *R*^2^ | 0.42 | 0.53 | 0.53 | 0.18 | 0.25 | 0.39 | 0.56 | 0.43 | 0.25 | 0.53 |

*Notes:* Standard errors in parentheses

^*^ *p* < 0.05, ^**^ *p* < 0.01, ^***^ *p* < 0.001
