# Supplementary material for: Manufacturing Epidemics: The Role of Global Producers in Increased Consumption of Unhealthy Commodities Including Processed Foods, Alcohol, and Tobacco
Source: PLoS Med. 2012 Jun 26;9(6):e1001235. doi: 10.1371/journal.pmed.1001235 (PMC3383750; doi:10.1371/journal.pmed.1001235)
Supplement: Text S7 — Free-trade agreements and soft drink consumption, 35 LMICs, year 2010. (DOC) [file pmed.1001235.s007.doc]

Supporting Information Text S7. Free-Trade Agreements and Soft Drink Consumption, 35 low- and middle-income countries, year 2010

|  | Soft Drink Consumption  (volume per capita) |
| --- | --- |
| 1% higher GDP per capita | 0.90%^***^ (0.19) |
| 1 percentage point higher urban population (% of total) | 0.018%^*^ (0.0068) |
| Free Trade Agreement with the United States | 55.4%^*^ (24.2) |
| Number of Countries | 35 |
| *R*^2^ | 0.74 |

^*^ *p* < 0.05, ^**^ *p* < 0.01, ^***^ *p* < 0.001
