# Supplementary material for: Manufacturing Epidemics: The Role of Global Producers in Increased Consumption of Unhealthy Commodities Including Processed Foods, Alcohol, and Tobacco
Source: PLoS Med. 2012 Jun 26;9(6):e1001235. doi: 10.1371/journal.pmed.1001235 (PMC3383750; doi:10.1371/journal.pmed.1001235)

Supporting Information Text S8. Association of Packaged Food Volume (per capita) with Sugar, Fat, and Salt Consumption per capita and Obesity and Diabetes Prevalence, 2005


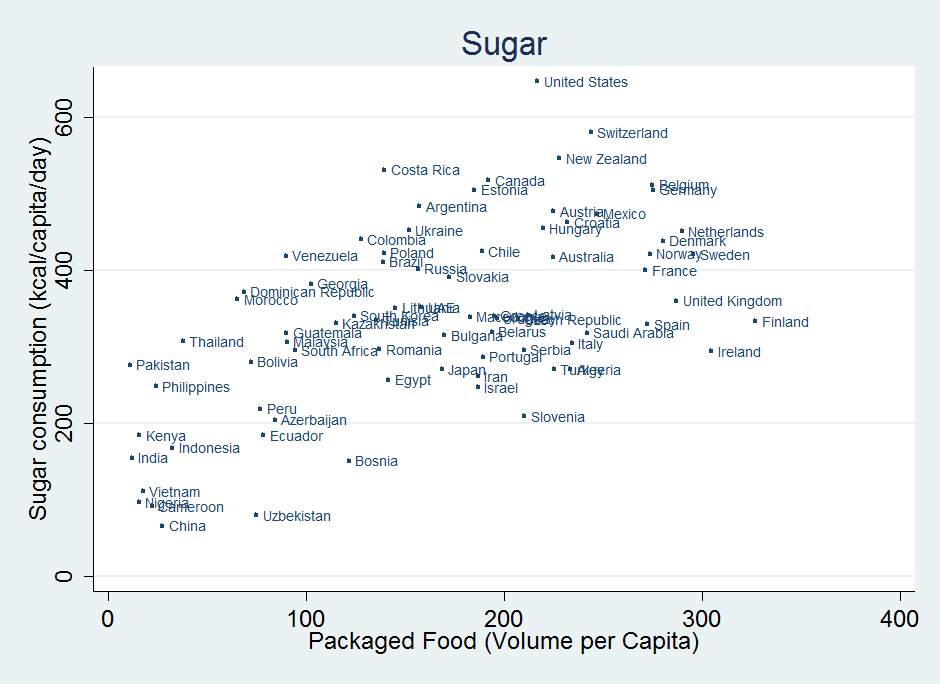

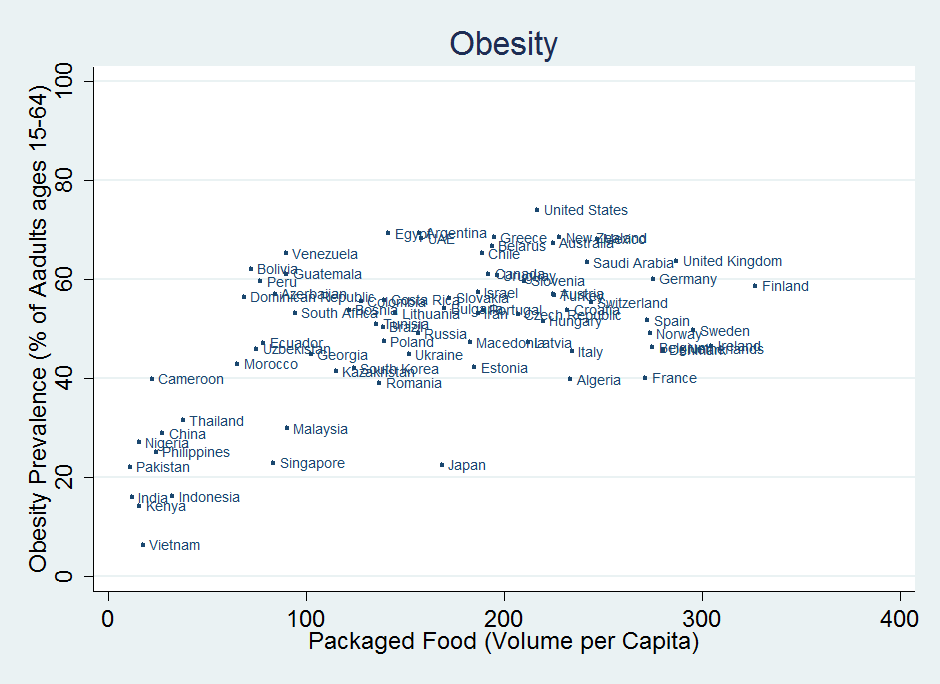

Supplement: Text S8 — Association of packaged food volume (per capita) with sugar, fat, and salt consumption per capita and obesity and diabetes prevalence, 2005. (DOC) [file pmed.1001235.s008.doc]
