# Supplementary material for: Manufacturing Epidemics: The Role of Global Producers in Increased Consumption of Unhealthy Commodities Including Processed Foods, Alcohol, and Tobacco
Source: PLoS Med. 2012 Jun 26;9(6):e1001235. doi: 10.1371/journal.pmed.1001235 (PMC3383750; doi:10.1371/journal.pmed.1001235)

Supporting Information Text S9. Three Population-Level Quasi-Natural Experiments of Soft Drinks, Tobacco, and Alcohol Consumption


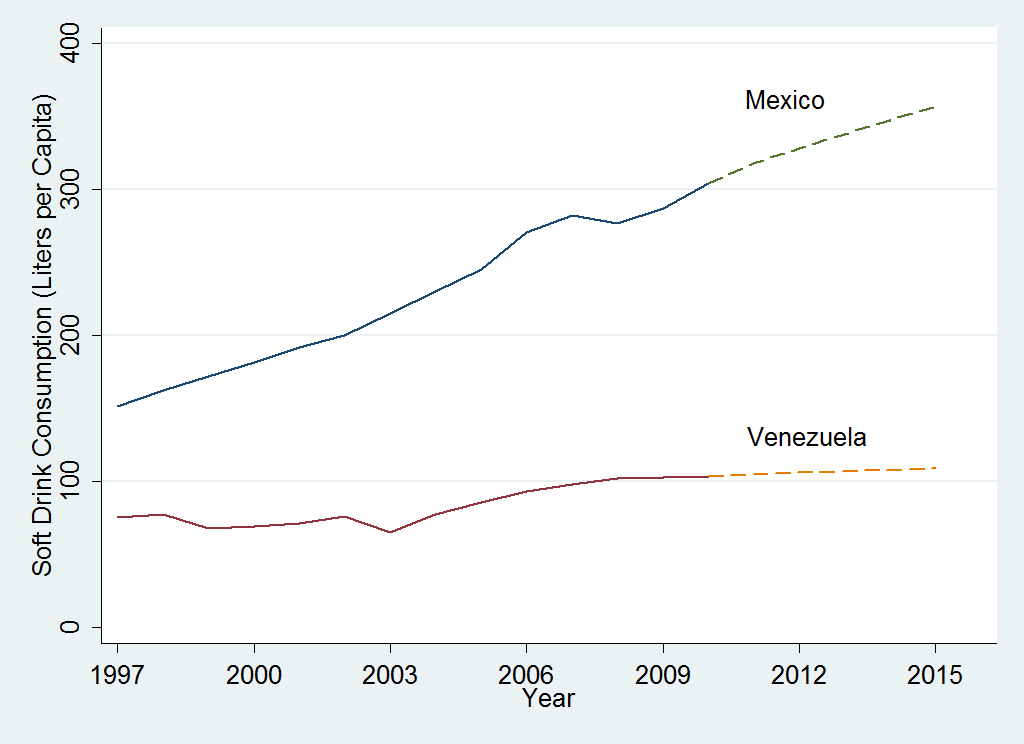

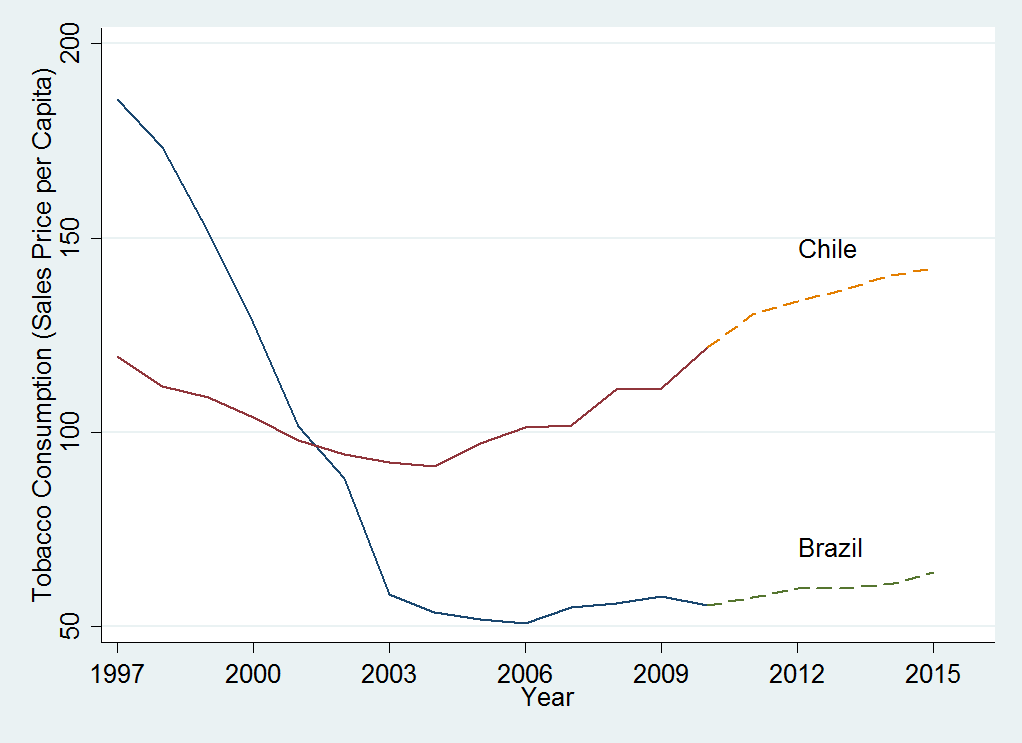


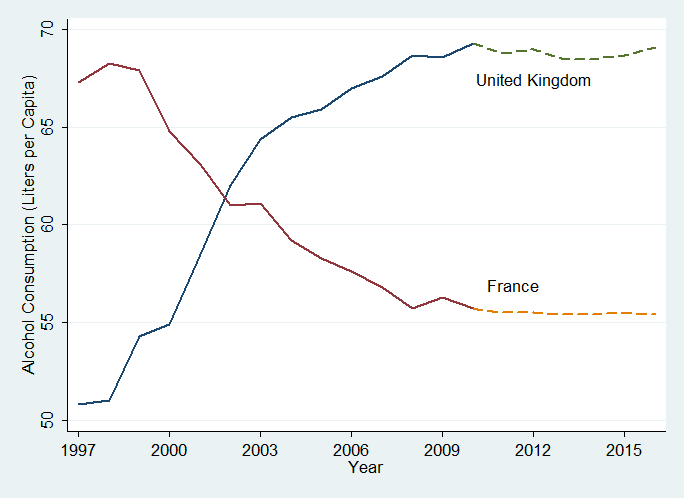

Supplement: Text S9 — Three population-level quasi-natural experiments of soft drinks, tobacco, and alcohol consumption. (DOC) [file pmed.1001235.s009.doc]
